# Evaluation of the clinical application value of cytokine expression profiles in the differential diagnosis of prostate cancer

**DOI:** 10.1007/s00262-024-03723-4

**Published:** 2024-06-04

**Authors:** Rongfa Chen, Linna Liu, Hui Chen, Chao Xing, Tingting Zhang, Yilin Pang, Xunjun Yang

**Affiliations:** 1 Department of Laboratory Medicine, The Second Affiliated Hospital and Yuying Children’s Hospital of Wenzhou Medical University, Wenzhou, 325000 Zhejiang China; 2 School of Laboratory Medicine and Life Sciences, Wenzhou Medical University, Wenzhou, 325035 Zhejiang China

**Keywords:** Prostatic Neoplasms, Cytokines, Blood, Peripheral, Luminex Technology

## Abstract

**Background:**

The significance of tumor-secreted cytokines in tumor development has gained substantial attention. Nevertheless, the precise role of tumor-related inflammatory cytokines in prostate cancer (PCa) remains ambiguous.

**Objectives:**

To gain deeper insights into the inflammatory response in the process of PCa.

**Methods:**

A total of 233 cases were collected, including 80 cases of prostate hyperplasia as disease control, 65 cases of postoperative prostate cancer and 36 cases of prostate cancer as PCa group. Additionally, 52 patients undergoing physical examinations during the same period were collected as the healthy control. The levels of 12 inflammatory cytokines in peripheral blood samples were analyzed using flow cytometric bead array technology. The levels of total prostate-specific antigen (TPSA) and free prostate-specific antigen (FPSA) in peripheral blood samples were analyzed using electrochemiluminescence technology.

**Results:**

Our findings revealed significant increases in serum IL-8 levels in PCa group compared to the healthy control group. Additionally, IL-6, IL-10, IFN-γ and IL-12p70 levels were markedly elevated in the PCa group compared to the disease control group (all *p* < 0.05). Conversely, the level of IL-4, TNF-α, IL-1β, IL-17A and IFN-α were lower in the PCa group compared to those in control group. Following surgery, the concentration of IL-6 decreased; whereas, the concentrations of IL-4, TNF-α, IL-17A, IL-1β, IL-12p70, and IFN-α increased, demonstrating significant differences *(p* < 0.05). The differential upregulation of IL-6 or downregulation of IL-17A in peripheral blood exhibited diagnostic efficacy in PCa patients. Moreover, we observed a significant increase in IL-17A levels, accompanied by decreased of IL-2, IL-4, IL-10, TNF-a, IFN-γ, IL-1β, and IL-12P70 in patients with distant metastasis.

**Conclusion:**

The peripheral blood cytokines are closely associated with the occurrence and development of prostate cancer, especially the serum levels of IL-6 and IL-17A may be useful as potential predictors of PCa diagnosis.

**Supplementary Information:**

The online version contains supplementary material available at 10.1007/s00262-024-03723-4.

## Introduction

Prostate diseases are prevalent worldwide and are among the most common diseases of the male genitourinary system. PCa is the second most common cancer in men globally, with a mortality rate second only to lung cancer [1, 2]. Currently, there are multiple theories and hypotheses regarding the pathogenesis of PCa, but the exact causes are still unclear. However, the occurrence and development of the disease are often accompanied by inflammation [3]. The activation and recruitment of immune cells during the cellular inflammatory response can lead to the enrichment of cytokines and chemokines in the tumor microenvironment, thereby affecting cancer development [4–6].

After infection, sentinel immune cells in the body recognize invading pathogens and release inflammatory mediators, including cytokines [7–9]. Therefore, inflammation can promote the occurrence and progression of tumors by affecting the body’s immune system [5]. Multiple studies have shown that Th17 cells are considered an important group of cells that mediate inflammatory responses, and the release of interleukin-17A (IL-17A) by Th17 cells contributes to the maintenance of chronic inflammatory states and the development of a cancer-promoting microenvironment, which is closely related to the occurrence and development of PCa [10–13]. In addition, recent studies have indicated that interleukin-6 (IL-6) and tumor necrosis factor-α (TNF-α) have different expressions in PCa tissues [14–16]. Another study found that interleukin-8 (IL-8) is also expressed in various cancers such as gastric cancer, esophageal cancer, and lung cancer to varying degrees [17–20]. Additionally, the remaining relevant reports include interleukin-2(IL-2), interleukin-4(IL-4), interleukin-5(IL-50), Interferon-α(IFN-α), Interferon-γ(IFN-γ), Interleukin-1β(IL-1β), and Interleukin-12p70(IL-12p70), all of which also associated with the occurrence and development of prostate disorders. IL-2 plays a critical role in immune responses, potentially influencing tumor development; IL-4, an anti-inflammatory cytokine, may regulate immune responses and tumor growth; IL-5 primarily regulates eosinophil proliferation, impacting tumor immune surveillance; IFN-α, widely studied in prostate disorders, enhances immune responses and inhibits tumor growth; IFN-γ, crucial for inflammation, influences prostate disorder progression; IL-1β, a pro-inflammatory cytokine, is implicated in tumor growth and metastasis as well as prostate disorders; IL-12p70 has been shown to inhibit prostate disorder growth and metastasis, suggesting its potential as an immunotherapy target.

These cytokines are complex responses stimulated during the tumor evolution process, and there have been few comprehensive reports on the comprehensive detection and analysis of cytokines in relation to the evolution of prostate cancer. Therefore, in this study, in order to better elucidate the relationship between prostate cancer and cytokines during the evolutionary process, we conducted a case–control study and systematically analyzed the concentration levels of cytokines in prostate-related diseases.

## Materials and methods

### Patients and specimens

This retrospective study received approval from the Ethics Committee of the Second Affiliated Hospital of Wenzhou Medical University, with ethics approval reference [No: 2021-K-301-01], and was conducted in accordance with the World Medical Association Declaration of Helsinki. The inclusion criteria were as follows: patients diagnosed and treated for prostate diseases at our hospital from January 2021 to April 2024, aged 30–90 years, and healthy individuals undergoing routine medical check-ups at the hospital during the same period. Exclusion criteria included underlying metabolic or infectious diseases, recent use of antibacterial drugs, history of other tumors, previous radiotherapy or chemotherapy. Additionally, patients with clear laboratory evidence of elevated levels of C-reactive protein (CRP), serum amyloid A (SAA), and procalcitonin (PCT), indicating signs of inflammation, were excluded.

### Diagnostic criteria for prostate diseases

According to the 2022 National Comprehensive Cancer Network (NCCN) guidelines, prostate cancer is diagnosed based on prostate biopsy, with a positive diagnosis indicating prostate cancer [21]; benign prostatic hyperplasia is diagnosed based on rectal examination indicating enlarged and firm prostate, ultrasound showing an enlarged prostate, increased residual urine, and decreased urine flow rate; urinary frequency, urgency, dysuria, turbid urine, or the presence of white secretions and pain in the lower abdomen indicate prostatitis.

### Sample collection

After standard aseptic procedures, 3 ml of residual serum from each participant was collected and immediately separated by centrifugation at 3000 rpm for 15 min, and the serum was stored in a − 80 °C freezer until further analysis.

### Quantitative detection of cytokines, TPSA, and FPSA

Flow cytometric bead array technology was used with a cytokine multiplex detection kit (Saiji(Jiangxi) Biotechnology Co., Ltd, China, 20,231,001) on a BD FACS CANTO II flow cytometer to detect the levels of IL-1β, IL-2, IL-4, IL-5, IL-6, IL-8, IL-10, IL-12p70, IL-17A, IFN-γ, IFN-α and TNF-α in peripheral blood samples. Electrochemiluminescence technology was used with a TPSA and FPSA Roche original detection kit on a Roche e601 electrochemiluminescence immunoassay analyzer (Roche (Shanghai) Co., Ltd., Switzerland) for detection.

### Statistical analysis

Continuous variables were presented as the median and interquartile range. Given the non-normal distribution of the data and a small sample size, nonparametric tests were chosen. The Mann–Whitney U-test was used to compare differences in serum cytokine levels, as well as age, blood WBC counts, TPSA, FPAS, etc., between controls and PCa. Similarly, the Mann–Whitney U-test was employed to compare serum cytokine levels among prostate cancer patients at different stages of development. Multiple Mann–Whitney tests were used to compare serum cytokine levels between preoperative and postoperative PCa patients. Spearman correlation coefficients were calculated for correlation analysis between serum cytokine levels in all participants. Statistical significance was set at *p* < 0.05. All analyses were conducted using SPSS version 20.0 (IBM Corp., Armonk, NY, USA) and GraphPad Prism 9.0 (GraphPad, San Diego, CA, USA). Plotting was performed using R (version 4.2.1) and ggplot2.

## Results

### Participant characteristics

**Fig. 1:**
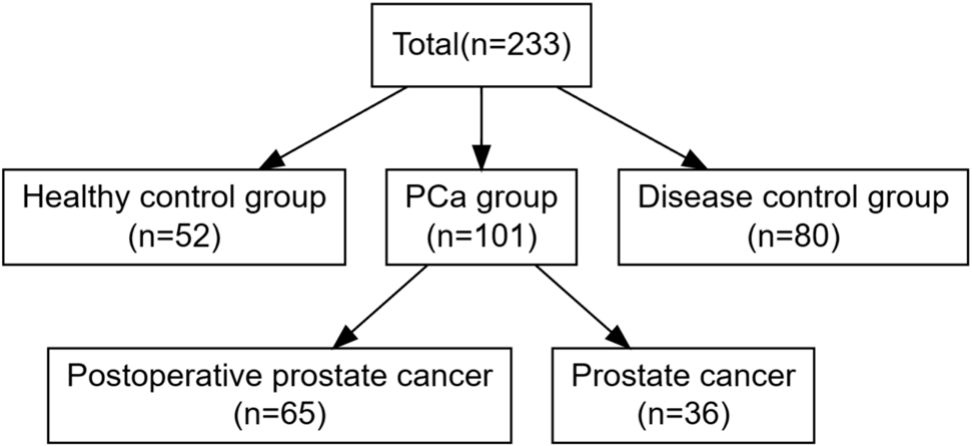
Consort diagram for the samples used in the study

A total of 233 participants were included in this study, and all patients informed consent, including 80 cases of prostate hyperplasia as disease controls, while 65 cases of postoperative prostate cancer and 36 cases of prostate cancer as PCa group. Additionally, 52 participants were collected as the healthy controls (Flow chart as seen in Fig. 1). The clinical information of all subjects as shown in Table 1. The participants were age-matched, and there were no differences in white blood cell and neutrophil levels, indicating the absence of inflammatory response in both groups. While, as specific tumor markers for prostate cancer, TPSA and FPSA showed significantly increased expression in the tumor group, which is consistent with the expected results.

**Table 1:** Clinical characteristics of the enrolled participant

Variables	Healthy control (*n* = 52)	Disease control (*n* = 80)	PCa group (*n* = 101)	*p* value
Age, years	66 (58.5–77.8)	68 (60–72)	68 (64–74)	0.447
WBC (*109/L)	6.12 (5.15–6.82)	6.19 (5.25–7.66)	6.28 (5.23–7.42)	0.680
Neutrophils (*109/L)	3.57 (3.10–4.14)	3.50 (2.82–5.18)	3.76 (3.03–4.73)	0.417
Lymphocyte (*109/L)	2.01 (1.65–2.34)	1.85 (1.40–2.11)	1.64 (1.22–2.07)	**0.003**
Monocytes (*109/L)	0.38 (0.30–0.44)	0.44 (0.36–0.57)	0.50 (0.42–0.63)	** < 0.0001**
Platelets (*109/L)	204 (182–240)	211 (171–234)	204 (168–241)	0.836
TPSA (ng/ml)	1.03 (0.63–1.50)	4.30 (1.37–6.62)	30.20 (6.77–194.5)	** < 0.0001**
FPSA (ng/ml)	0.28 (0.19–0.39)	0.74 (0.35–1.40)	4.49 (1.24–20.6)	** < 0.0001**

Values are given as median (interquartile range), WBC, means white blood cell

Bold indicates statistical difference (*p* < 0.05)

### Cytokines characteristics in the participant

**Fig. 2:**
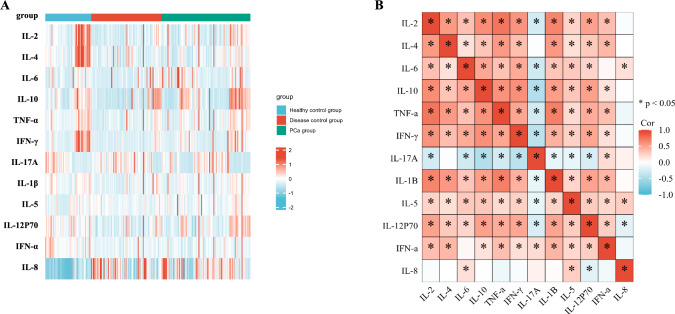
Concentrations of cytokines in patients with PCa and control groups. **A** Heatmap of differentially expressed 12 serum multi-cytokines between participants with PCa patients and non-tumor patients (disease and healthy controls) using Luminex assay. **B** Correlation heatmap of 12 serum cytokines in all participant.

According to the hospital-recommended program, 12 candidate serum cytokines were selected for analysis. The heatmap displays the concentrations of these cytokines in the participants (Fig. 2A). The correlation heatmap showed their correlation between the 12 cytokines (Fig. 2B).

### The distribution of cytokines in the peripheral blood of PCa patients

**Fig. 3:**
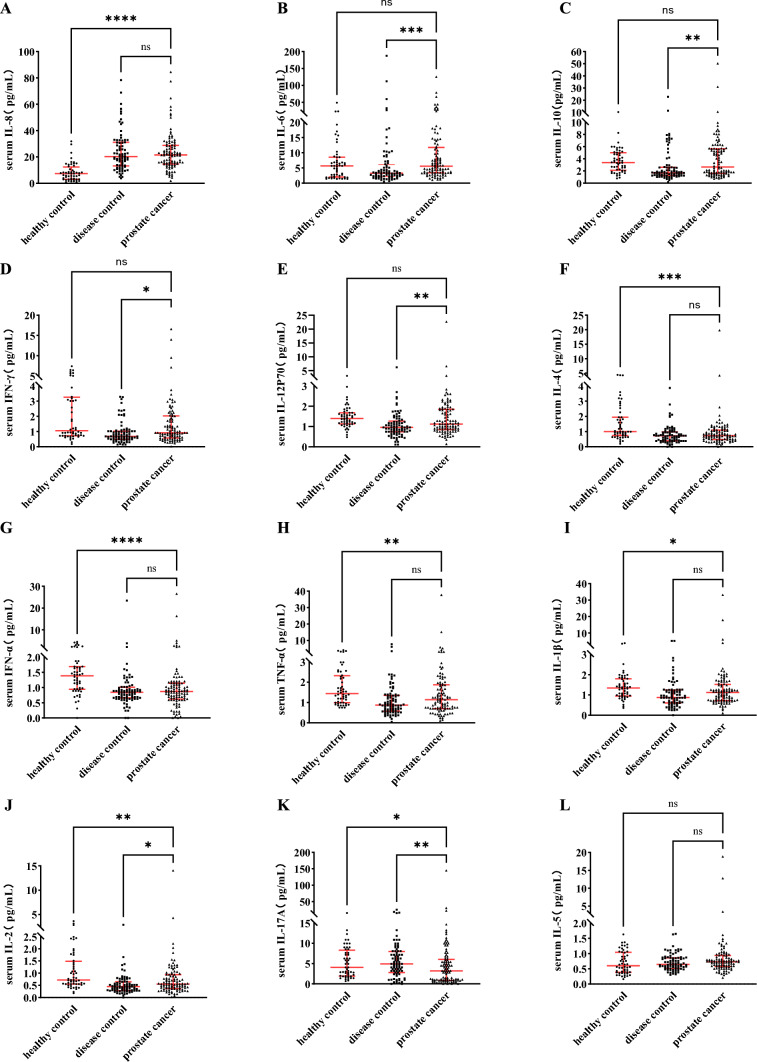
Distribution of cytokines in the peripheral blood of participant. The levels of IL-8 (**A**), IL-6(**B**), IL-10 (**C**), IFN-γ (**D**), IL-12p70 (**E**), IL-4 (**F**), INF-α (**G**), TNF-α (**H**), IL-1β (**I**), IL-2 (**J**), IL-17A (**K**), IL-5 (**L**) in serum from healthy and disease controls and PCa group. The red horizontal lines show the medians and interquartile range. The difference between control and PCa was assessed using the Mann–Whitney U-test.

To compared the distribution of cytokines between PCa and the control group. The result as shown in Fig. 3, The levels of pro-inflammatory cytokines IL-8 were significantly increased in the peripheral blood of patients with PCa than in the healthy control group; while, IL-6, IL-10, IFN-γ and IL-12p70 were higher in patients with PCa than in the disease control group (all *p* < 0.05). There was no difference in the levels of IL-5 between the three groups (all *p* > 0.05). The surprising discovery is in the prostate cancer group, the concentrations of the inflammatory cytokines IL-4, TNF-α, IL-1β, and IFN-α were significantly lower than those in the healthy control group (all *p* < 0.05). The detail data shown in Table 1S.

### The difference in serum cytokine distribution between patients undergoing prostate cancer surgery and those who have not undergone surgery

**Fig. 4:**
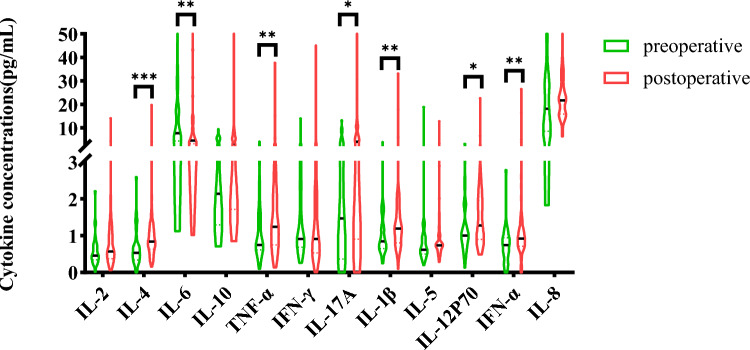
Relationship between distribution of peripheral blood cytokines and prostate cancer surgery in PCa patients

To analyze whether cytokines are associated with the surgical prognosis of prostate cancer patients, we compared the distribution of cytokines between prostate cancer patients and postoperative patients. In addition, we performed a more detailed analysis of the clinical data of PCa patients between these groups, and there were no significant differences in age, sex, BMI category, tumor location, tumor histology, and tumor size. Figure 4 shows the concentration of IL-6 decreased after surgery, showing a significant difference (*p* < 0.05). The concentrations of IL-4, TNF-α, IL-17A, IL-1β, IL-12P70 and IFN-α increased after surgery, showing significant differences (*p* < 0.05); while, the concentrations of the other cytokines showed no significant difference (all *p* > 0.05). In this part of the analysis indicating that these cytokines are influenced by the excision of the primary tumor.

### Serum cytokines as possible diagnostic biomarkers for prostate cancer

**Fig. 5:**
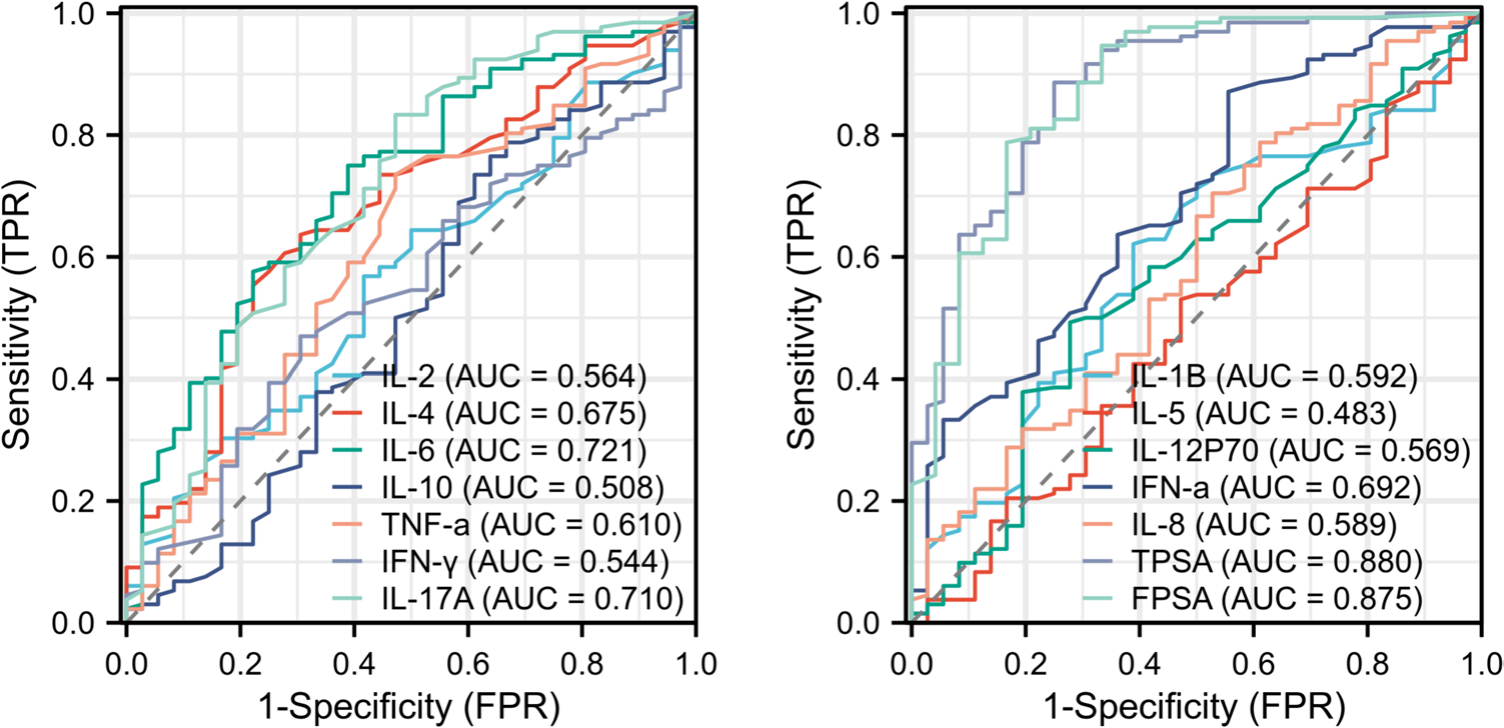
Schematic illustration of ROC curve to evaluate the diagnostic potential of serum IL-6, IL-8 and IL-5 to differentiate PCa group from controls

To confirm the preceding findings, we performed ROC analysis. Figure 5 shows the ROC of the presence of PCa when cytokines were used. The area under the curve (AUC) of IL-6 and IL-17A in the diagnosis of prostate cancer patients were 0.721, and 0.710, respectively, demonstrates “good” capability for discriminating between patients with PCa and controls. Positive and negative predictive value, sensitivity and specificity of each cytokine’s diagnosis in PCa show in Table 2S.

### The relationship between cytokine levels and metastasis, Gleason Score of prostate cancer patients

To further investigate the relevance of cytokines in prostate cancer development, we compared the distribution of various cytokines in the serum of patients with different Gleason scores and those with metastasis. The results revealed no statistically significant differences in cytokine distribution among prostate cancer patients with different Gleason scores (see in Table 3S). However, in patients with distant metastasis of prostate cancer, we found a significant increase in IL-17A levels; while, levels of cytokines IL-2, IL-4, IL-10, TNF-a, IFN-γ, IL-1β, and IL-12P70 were significantly decreased (As show in Table 2). This suggests that cytokines may contribute to the metastatic process of prostate cancer.

**Table 2:** Relationship between cytokine levels and metastasis of prostate cancer patients

Cytokines	Metastasis		*p* value
	No (*n* = 63)	Yes (*n* = 18)	
IL-2	0.68 (0.42–1.02)	0.41 (0.29–0.55)	**0.014**
IL-4	0.82 (0.55–1.15)	0.53 (0.3–0.79)	**0.003**
IL-6	5.14 (3.39–8.91)	6.73(3.75–18.02)	0.110
IL-10	4.91 (1.82–6.31)	1.88 (1.26–2.72)	**0.001**
TNF-a	1.24 (0.8–2.2)	0.75 (0.47–1.1)	**0.003**
IFN-γ	1.33 (0.65–2.65)	0.72 (0.51–0.96)	**0.005**
IL-17A	1.24 (0.79–5.38)	4.94 (2.9–9.13)	**0.017**
IL-1β	1.22 (0.83–1.72)	0.74 (0.58–1.12)	**0.000**
IL-5	0.75 (0.58–0.94)	0.69 (0.62–0.82)	0.482
IL-12P70	1.48 (0.9–2.06)	0.82 (0.65–1.16)	**0.001**
IFN-a	0.87 (0.66–1.2)	0.86 (0.61–1.01)	0.576
IL-8	20.5 (13.95–27.6)	23.68 (15.87–31.23)	0.072

Bold indicates statistical difference (*p* < 0.05)

## Discussion

PCa stands as the most prevalent malignant tumor among males and ranks as the fifth leading cause of cancer-related death globally [22]. Inflammation plays a key role in the occurrence and development of tumors, and prostate cancer is characterized by excessive expression of inflammation. Inflammatory cytokines are essential in the development of diseases into tumors and can directly act on tumor cells or indirectly exert multifaceted effects through the tumor microenvironment, promoting cancer cell proliferation [4–6].

Cytokines are small protein molecules secreted by various cells in the body and have a wide range of biological activities. They can be divided into pro-inflammatory and anti-inflammatory cytokines. Pro-inflammatory cytokines primarily secreted by lymphocytes and monocytes include IL-1β, IL-2, IL-5, IL-6, IL-8, IL-12p70, IL-17A, IFN-γ, IFN-α, and TNF-α. Anti-inflammatory cytokines include IL-4 and IL-10 [23–25].

In our study, after comprehensive analysis of 12 cytokines involved in prostate cancer and related diseases, we observed significant differences in many serum cytokine levels between prostate cancer patients and the control group. Specifically, concentrations of the inflammatory cytokines IL-8, IL-6, IL-10, INF-γ and IL-12P70, were significantly increase in the prostate cancer group compared to the control group; while, the concentrations of IL-4, IFN-α, TNF-α and IL-1β were significantly lower than those in the control group. Paul Katongole and others also found increased IL-6 levels in the serum of prostate cancer patients [26]. Additionally, other cytokines such as TNF-α, IL-8, and IL-1β have also been found to be associated with the development and progression of prostate cancer [27]. These cytokines can affect the growth and metastasis of prostate cancer by regulating inflammation, angiogenesis, and tumor cell proliferation in the tumor microenvironment. These cytokines are mainly secreted by T and B lymphocytes and are important components of the immune response, playing important roles in various biological functions, including immune response against bacterial infections [28]. These results indicate that inflammation often occurs during the evolution of prostate cancer and these indicators may be essential in diagnosing the disease.

Our study revealed a significant elevation in IL-6 concentration within the prostate cancer group compared to the disease control group, indicating a potential involvement of IL-6 in prostate cancer progression. This observation aligns with prior research documenting heightened IL-6 levels in the peripheral blood of individuals with prostate cancer [29–31]. IL-6 is recognized for its pivotal role in prostate-mediated immune responses, exerting influence on both humoral and cellular immune responses. It acts on B and T lymphocytes, stimulating their growth and differentiation, and contributes to inflammation induced by follicular Th cells [28, 29, 32].

Furthermore, we analyzed the changes in cytokine concentrations before and after prostate cancer surgery. We found that the concentration of IL-6 decreased after surgery; while, the concentrations of IL-4, TNF-α, IL-17A, IL-1β, IL-12P70 and IFN-α increased significantly. These findings suggest that surgery may have an impact on the inflammatory response and cytokine levels in prostate cancer patients. In other words, it is possible that the presence of the tumor leads to the continuous production of certain cytokines.

Additionally, our present data provide preliminary support for utilizing differential levels of IL-6 and IL-17A as indicators of existing PCa. Emerging evidence suggests that chemokines and cytokines are responsible for the pleiotropic actions in cancer, including the growth, angiogenesis, endothelial mesenchymal transition, leukocyte infiltration, and hormone escape for advanced PCa and therapy resistance. Previous studies have indicated that Melittin inhibits tumor cell migration and enhances cisplatin sensitivity by suppressing IL-17 signaling pathway gene LCN2 in castration-resistant prostate cancer [33].

Moreover, we have also found that multiple cytokines are correlated with the metastasis of prostate cancer. The literature on the association between cytokines and prostate cancer metastasis is quite extensive [34]. These studies typically involve measuring cytokine levels in patient serum or tissue samples and analyzing the relationship between these factors and prostate cancer metastasis and prognosis. Some studies also explore the role and mechanisms of specific cytokines in the pathway of prostate cancer metastasis [35]. These studies suggested cytokines hold significant potential in both the development of prostate cancer and its clinical monitoring.

Our study also has certain limitations. Firstly, although there is a biological relationship between inflammatory cytokines and prostate cancer and they have some statistical significance, our observations may have certain limitations. Before these results can be widely applied in clinical settings, it is necessary for us to repeatedly validate these conclusions by increasing the size of our research cohort. Secondly, our samples were collected at the same time point, and the cytokine concentrations were measured at a single time point, potentially overlooking potential changes over time. Thirdly, the relative imbalance in the collected samples across different groups may introduce certain biases to the conclusions.

In conclusion, our study provides insights into the distribution of peripheral blood cytokines in prostate cancer and related diseases, offering laboratory evidence for the relationship between disease occurrence, development, and prognosis. The changes in cytokine levels after prostate cancer surgery may also serve as targets for treatment. Nonetheless, our results contribute to the understanding of the role of cytokines in prostate cancer and provide potential avenues for further research and clinical applications.

### Supplementary Information

Below is the link to the electronic supplementary material.

Supplementary file1 (DOCX 26 KB)
