# Supplementary material for: Evaluation of the clinical application value of cytokine expression profiles in the differential diagnosis of prostate cancer
Source: Cancer Immunol Immunother. 2024 Jun 4;73(8):139. doi: 10.1007/s00262-024-03723-4 (PMC11150366; doi:10.1007/s00262-024-03723-4)
Supplement: Supplementary file 1 — Supplementary file1 (DOCX 26 KB) [file 262_2024_3723_MOESM1_ESM.docx]

**Table S1Analysis of differential distribution of cytokines among different groups**

| **Cytokine** | **Healthy control**  **(n=52)** | **Disease control**  **(n=80)** | **PCa group**  **(n=101)** | ***p* value** | |
| --- | --- | --- | --- | --- | --- |
| IL-2 | 0.72(0.55-1.49） | 0.45(0.29-0.65） | 0.55(0.36-0.94） | | **.000** |
| IL-4 | 1(0.74-1.94） | 0.72(0.39-0.98） | 0.73(0.48-1.1） | | **.000** |
| IL-6 | 5.67(2.01-8.53） | 3.25(2.17-6.1） | 5.55(3.39-11.73） | | **.001** |
| IL-10 | 3.38(2.12-5） | 1.68(1.25-2.6） | 2.65(1.61-5.65） | | **.000** |
| TNF-a | 1.44(0.99-2.33） | 0.87(0.57-1.35） | 1.14(0.7-1.88） | | **.000** |
| IFN-γ | 1.06(0.72-3.26） | 0.68(0.49-1.02） | 0.91(0.57-2.03） | | **.000** |
| IL-17A | 4.07(1.95-8.32） | 4.94(2.8-7.98） | 3.21(0.84-6.05） | | **.005** |
| IL-1β | 1.35(0.95-1.79） | 0.88(0.61-1.25） | 1.12(0.72-1.52） | | **.000** |
| IL-5 | 0.6(0.38-1.04） | 0.65(0.51-0.86） | 0.73(0.57-0.94） | | .132 |
| IL-12P70 | 1.4(1.13-1.68） | 0.96(0.74-1.27） | 1.12(0.85-1.85） | | **.000** |
| IFN-a | 1.38(0.95-1.69） | 0.84(0.66-1.01） | 0.87(0.62-1.14） | | **.000** |
| IL-8 | 7.38(3.03-12.35） | 20.33(13.22-31.11） | 21.58(14.63-28.79） | | **.000** |

Note: Values are given as median (interquartile range), Bold indicates statistical differences

**Table 2S The specific statistical data for diagnosing ROC curves**

| **Cytokines** | **Youden index** | **AUC** | **cut-off** | **sensitivity** | **specificity** | **Positive predictive** | **negative predictive** |
| --- | --- | --- | --- | --- | --- | --- | --- |
| IL-2 | 0.15 | 0.56 | 0.50 | 0.57 | 0.58 | 0.83 | 0.27 |
| IL-4 | 0.33 | 0.68 | 0.69 | 0.64 | 0.69 | 0.88 | 0.34 |
| **IL-6** | **0.36** | **0.72** | **6.97** | **0.75** | **0.61** | **0.88** | **0.40** |
| IL-10 | 0.13 | 0.51 | 4.37 | 0.77 | 0.36 | 0.81 | 0.30 |
| TNF-a | 0.26 | 0.61 | 0.77 | 0.73 | 0.53 | 0.85 | 0.35 |
| IFN-γ | 0.16 | 0.54 | 0.76 | 0.47 | 0.69 | 0.85 | 0.26 |
| **IL-17A** | **0.36** | **0.71** | **1.53** | **0.83** | **0.53** | **0.87** | **0.46** |
| IL-1B | 0.23 | 0.59 | 0.90 | 0.62 | 0.61 | 0.85 | 0.31 |
| IL-5 | 0.06 | 0.48 | 0.63 | 0.53 | 0.53 | 0.80 | 0.23 |
| IL-12P70 | 0.21 | 0.57 | 1.16 | 0.49 | 0.72 | 0.87 | 0.28 |
| IFN-a | 0.32 | 0.69 | 0.61 | 0.87 | 0.44 | 0.85 | 0.48 |
| IL-8 | 0.18 | 0.59 | 21.93 | 0.70 | 0.47 | 0.83 | 0.30 |
| **TPSA** | **0.64** | **0.88** | **8.26** | **0.89** | **0.75** | **0.93** | **0.64** |
| **FPSA** | **0.62** | **0.88** | **1.08** | **0.79** | **0.83** | **0.96** | **0.42** |

Note: Bold indicates better diagnostic performances

**Table 3S The relationship between cytokine levels and Gleason score of PCa**

| **Cytokines** | **Gleason Score** | | | ***p* value** |
| --- | --- | --- | --- | --- |
|  | **≦6（n=18）** | **7（n=18）** | **≥8（n=41）** |  |
| IL-2 | 0.53(0.36-1.04） | 0.55(0.32-0.96） | 0.55(0.35-0.89） | 0.578 |
| IL-4 | 0.89(0.66-1.15） | 0.62(0.43-0.82） | 0.69(0.49-1.14） | 0.213 |
| IL-6 | 4.6(3.11-5.93） | 6.22(3.66-11.9） | 7.11(3.48-14.48） | 0.112 |
| IL-10 | 2.62(1.69-5.58） | 4.29(2.36-6.05） | 2.42(1.54-5.93） | 0.244 |
| TNF-a | 1.22(0.79-2.26） | 1.18(0.64-2.49） | 1.07(0.7-1.8） | 0.594 |
| IFN-γ | 1.53(0.8-4.23） | 1.01(0.75-2.16） | 0.91(0.46-2.03） | 0.476 |
| IL-17A | 3.61(0.75-7.87） | 2.16(0.66-7.07） | 2.8(0.94-5.38） | 0.760 |
| IL-1β | 1.16(0.71-1.61） | 0.91(0.68-2.02） | 1.19(0.8-1.42） | 0.890 |
| IL-5 | 0.78(0.72-0.95） | 0.68(0.5-0.8） | 0.73(0.62-0.98） | 0.796 |
| IL-12P70 | 1.49(0.98-1.98） | 1.16(0.9-1.86） | 1.13(0.75-2.03） | 0.244 |
| IFN-a | 0.84(0.65-1.04） | 0.84(0.45-1.23） | 0.87(0.68-1.14） | 0.890 |
| IL-8 | 22.18(15.26-29.91） | 19.24(11.99-25.53） | 21.68(15.26-29.7） | 0.570 |
